# Epithelioma—Blepharoplasty

**Published:** 1879-02

**Authors:** 


### ARTICLE VII.

### *Epithelioma—Blepharoplasty.*

Dr. Noyes, at a recent meeting of the New York Pathological Society, exhibited a small specimen of epithelial growth removed by operation from the eyelids, which was of interest in connection with the means used to fill up the gap which was left. The patient was a lady aged 50 years. Nineteen years ago she noticed a small growth on the inner border of the lower lid, near the punctum. This remained stationary for ten or twelve years, when it began to spread along the border of the lid and at the same time broke down in ulceration. Within the past year the disease extended itself over the lachrymal sac, involved the inner portion of the upper lid, and made in all a tumor the size of a hazelnut. Two-thirds of the lower and one-third of the upper lid were occupied by the disease as well as the adjoining side of the nose. The operation of removal was performed without difficulty, the tumor shelling out from the underlying tissues easily. The lachrymal sac was exposed, but was not opened. Actual cautery was employed along the inner portion of the wound. In order to fill up the deficiency of the growth occasioned by the extirpation, a vertical incision was first made in a line with the inner canthus and along the side of the nose to the reflection of the mucous membrane of the gum. From the outer canthus an incision was also extended horizontally across the malar bone to within a half inch of the ear. The flap thus created was dissected up and slid inward toward the nose, thus restoring the lower lid. A space was left on inner canthus and inner portion of upper lid. An attempt was made to fill up this space by transplanting a severed portion of skin from the arm, according to the method proposed by Mr. Wolf, of Glasgow. But sloughing of this piece occurred, owing, as Dr. Noyes thought, to the fact that actual cautery had been previously used in that portion of the bed of the wound. Union occurred by first intention throughout the flap for the lower eyelid. A second attempt to fill up the vacancy of inner portion of upper lid was made shortly afterward. An incision from the upper limit of the gap was made horizontally across the root of the nose, and then at right angles downward. The flap thus created was turned upon itself edgewise and accurately adapted to the edges of the gap. Her recovery was complete and satisfactory. The tumor was examined by Drs. Bull and Satterthwaite, and found to be an epithelioma.—*Med. Record.*

---

